# Implementation of a Night Service of Helicopter Transportation to Reduce the Time to Revascularization in STEMI Patients in a Mountainous Region: Impact on Outcomes

**DOI:** 10.3390/jcm11175089

**Published:** 2022-08-30

**Authors:** Filippo Zilio, Marta Rigoni, Simone Muraglia, Marco Borghesi, Federico Zucchelli, Daniel Todaro, Michele Dallago, Giuseppe Braito, Fabrizio Damaggio, Giandomenico Nollo, Roberto Bonmassari

**Affiliations:** 1 Department of Cardiology, Santa Chiara Hospital, 38122 Trento, Italy; 2 BIOtech Lab, Department of Industrial Engineering, University of Trento, 38123 Trento, Italy; 3 Department of Biomedical, Surgical and Dental Sciences, University of Milan, 20122 Milan, Italy

**Keywords:** STEMI, STEMI network, time to revascularization

## Abstract

Background: Treatment delays are the most easily audited index of quality of care in the setting of ST-segment elevation myocardial infarction; among the components of ischemia time, system delay has been demonstrated to be a predictor of outcomes, and in a mountainous region it relies mostly upon helicopter rescue service. Aim: The aim of the study is to analyze the impact of the activation of helicopter rescue service for the nighttime for urgent transportation of patients on the time to revascularization and on the outcomes of STEMI patients. Methods: Data were prospectively collected in a database and retrospectively split into two different cohorts, based on the presentation date in the 18 months before, or after, the first day of implementation of the new organizational model. The patients were also split into two groups based on the place of STEMI diagnosis, either the chief town territory or the rest of the region, and retrospectively evaluated for vital status at 30 days and 2 years after index event. Results: The number of patients included was 751. For patients coming from outside Trento, an improvement in ST-segment resolution was shown (ST-segment elevation reduction >50% in 54.0% of the patients vs. 36.4%, *p* < 0.01). Moreover, a reduction in diagnosis-to-reperfusion median time has been demonstrated (from 105 to 97 min, *p* < 0.01), mainly driven by a reduction during the night shift (from 119 to 100 min, *p* = 0.02). With regard to 30-day and 2-year mortality, no statistically significant differences were achieved. Discussion: The organizational effort has translated into a significant reduction in the treatment delay for patients coming from outside the chief town. However, although a longer diagnosis to reperfusion time has been related to a higher mortality, a significant reduction in mortality was not demonstrated in our study. However, an improvement in ST-segment elevation resolution was shown for patients coming from outside the city of Trento, a result that could have other potential clinical benefits. Conclusions: Implementation of night flight proved to be effective in reducing the time between the diagnosis and the treatment of patients in the setting of STEMI, improving ST-segment elevation resolution, although no impact was shown on short- and long-term mortality.

## 1. Introduction

Ischemic heart disease is the most common cause of death in the world and it accounts for about 20% of all the deaths in Europe [1]. Among the manifestations of this disease, the incidence rate of ST-segment elevation myocardial infarction (STEMI) ranged from 43 to 144 per 100,000 per year in registries from Europe and the USA [2,3,4]. 

Many factors influence the mortality in STEMI patients, such as advanced age, Killip class, time delay to treatment and presence of emergency medical system (EMS)-based STEMI networks, treatment strategy, history of myocardial infarction (MI), diabetes mellitus, renal failure, number of diseased coronary arteries, and left ventricular ejection fraction (LVEF) [5]. Among the factors influencing mortality, reperfusion therapy with primary percutaneous coronary intervention (PCI) has been reported as one of the determinants of the fall in 1-year mortality among STEMI patients to approximately 10% [1,6,7,8,9] and it is now the gold-standard treatment for patients affected by STEMI [5].

However, as stated in the most recent European Society of Cardiology (ESC) Guidelines on the management of acute myocardial infarction in patients presenting with ST-segment elevation, treatment delays are the most easily audited index of quality of care in STEMI. To ensure the meeting of that simple quality of care indicator, the timing of the components of ischemia time should be recorded in every system providing care to STEMI patients and reviewed regularly [5]. While patient delay is not readily modifiable, reduction in system delay should be addressed to reduce total ischemic time; system delay, indeed, has been demonstrated to be a predictor of outcomes [10]. Target delay for implementation of primary PCI as a quality indicator is wire crossing of infarct-related artery in less than 90 min after STEMI diagnosis, or in less than 60 min if the patient presents directly to a PCI-capable hospital, while 120 min is the cut-off to prefer PCI over fibrinolysis [5]. Although no significant reduction in 30-day in-hospital mortality has been demonstrated in a large-scale multicenter study for STEMI patients whose diagnosis-to-reperfusion (DTR) time was ≤90 min, improvement of DTR time should still be persistently emphasized because of a potential benefit in other crucial endpoints such as long-term mortality, left ventricular function, or heart failure [11]. With this aim, different strategies have been developed to reduce DTR time, including direct activation of the catheterization laboratory, completion of PCI team preparation within 20–30 min after the call, rapid data feedback, adoption of a team-based approach, and administrative support [12]. A recent study, for example, showed a 1 min improvement in DTR time by implementing a chief complaint-based “cardiac triage” protocol [13]. However, other strategies should be evaluated to achieve a reduction in DTR time.

The Province of Trento is a mountainous region of Italy, extending for 6206.87 km^2^, with 538,223 inhabitants. Since 2011, a “hub and spoke” model has been officially established for STEMI patients, with a single 24/7 catheterization laboratory located in the chief town of Trento and six spoke hospitals connected with ambulance and helicopter rescue service, due to the orography of the region. Although a significant investment of resources was required, since 1 July 2013, night flight of helicopters was implemented, with the aim of reducing the time from first medical contact to wire crossing of the lesion for STEMI patients calling the EMS from outside the chief town territory or presenting to spoke hospitals after sunset.

## 2. Aim

The aim of the study is to analyze the impact of the activation of helicopter rescue service for the nighttime for urgent transportation of patients on the time to revascularization and on the outcomes of STEMI patients. To evaluate the efficacy of the new organizational model, we compared the time required for patient management for 18 months before and after the implementation date, together with procedural result parameters and performance indicators.

## 3. Methods

In this longitudinal study, clinical and angiographic data of consecutive patients who underwent primary PCI for STEMI at our facility in Trento (Italy) from 1 January 2012 to 31 December 2014 were prospectively collected in a registry, together with time of first medical contact (either as ECG diagnosis of STEMI by the EMS or as hospital presentation) and time of revascularization (wiring) of infarct-related lesion. For patients for whom PCI was not advised, either because of absence of obstructive coronary artery disease or because of anatomical considerations leading to indication to coronary artery bypass grafting (CABG) or to medical-only treatment, time of conclusion of the diagnostic coronary angiography was taken into consideration.

Cardiovascular risk factors and clinical and hemodynamic characteristics were collected for each patient in an electronic database. Hypertension was defined as systolic pressure >140 mmHg and/or diastolic pressure >90 mmHg or if the individual was taking antihypertensive medications. The diagnosis of diabetes was based on previous history of diabetes treated with or without drug therapies, or fasting glucose >126 g/dL or HbA1c > 6.5% at the moment of admission. Chronic renal failure was considered for history of renal failure or an admission glomerular filtration rate (GFR) < 60 mL/min/1.73 m^2^ by the Modification of Diet in Renal Disease (MDRD) formula.

The study was reviewed and approved by the Ethics Committee of “Azienda Provinciale per i Servizi Sanitari” (APSS) of Trento (Project identification code: RETE STEMI) and managed in accordance with Good Clinical Practice and the Declaration of Helsinki. The need to obtain informed consent from participants was waived owing to the retrospective nature of the study.

In 2020, the patients were retrospectively split into 2 different cohorts, based on the presentation date before, or after, the 1st of July, 2013. Since that day, in fact, a new protocol was implemented with regard to pre-hospital and inter-hospital transport of patients affected by STEMI, with extension of the helicopter rescue service to the nighttime. The patients were also split into 2 groups based on the place of STEMI diagnosis, either the chief town territory or the rest of the region.

Primary outcome of the study was the diagnosis to reperfusion (DTR) time. Secondary outcomes were ST-segment elevation reduction >50%, final TIMI flow 3, and mortality at 30 days and 2 years after index event.

Sample size calculation was not performed, since we wanted to include the largest number of patients.

Continuous variables were checked for normality with a Shapiro–Wilk test, and were expressed as mean and standard deviation (SD) or median and first and third quartiles (Q1–Q3), according to the normality of the distribution. Categorical variables and scores were expressed as numbers and percentages. Baseline characteristics and clinical outcomes between groups were compared with chi-square, Fisher’s exact, Student’s *t*, and Mann–Whitney U tests, as appropriate. Survival analysis was performed using a univariable Cox proportional hazard model, differences between groups were assessed with a log-rank test, and graphs were made with the Kaplan–Meier method. A p-value less than 0.05 indicated statistical significance. All analyses were performed with Stata statistical software (Stata Corp, College Station, TX 77845, USA).

## 4. Results

The number of patients treated with primary PCI for STEMI during the overall period was 751. To evaluate the impact of the different organizational models, we separately analyzed data of patients for whom the diagnosis of STEMI was given in the chief town of Trento territory (either by the EMS or in the Emergency Department), and data of patients whose STEMI diagnosis was given elsewhere in the region, either by the EMS or in a spoke hospital.

Baseline characteristics of the subgroups of patients are reported in Table 1; procedural results and performance indicators are reported in Table 2. In the two groups, there were no differences with regard to clinical, procedural, and logistical baseline characteristics before and after the implementation of the new organizational model, except for a higher percentage of patients transported to the hospital from outside Trento by helicopter in the second time frame, as expected.

With regard to procedural results (successful PCI, TIMI 3 flow restoration, ST-segment elevation resolution at the post-procedural ECG) and performance indicators (median DTR time, percentage of patients treated within 90 min and within 120 min from STEMI diagnosis), no significant differences were detected for patients evaluated in the urban setting which showed a non-statistical increase in DTR median time after 1 July 2013 (from 68 to 75 min, *p* = 0.07). For patients coming from outside Trento, an improvement in ST-segment resolution was shown (ST-segment elevation reduction >50% in 54.0% of patients vs. 36.4%, *p* < 0.01). Moreover, a reduction in DTR median time has been demonstrated (from 105 to 97 min, *p* < 0.01), mainly driven by a reduction during the night shift (from 119 to 100 min, *p* = 0.02). Coherently, the goal of a DTR time ≤120 min was obtained in 76.9% of patients vs. 64.3% (*p* < 0.01); however, a DTR time ≤90 min was obtained in 40.9% vs. 32.9% of patients, an increase that was not significant (*p* = 0.09).

**Figure 1:**
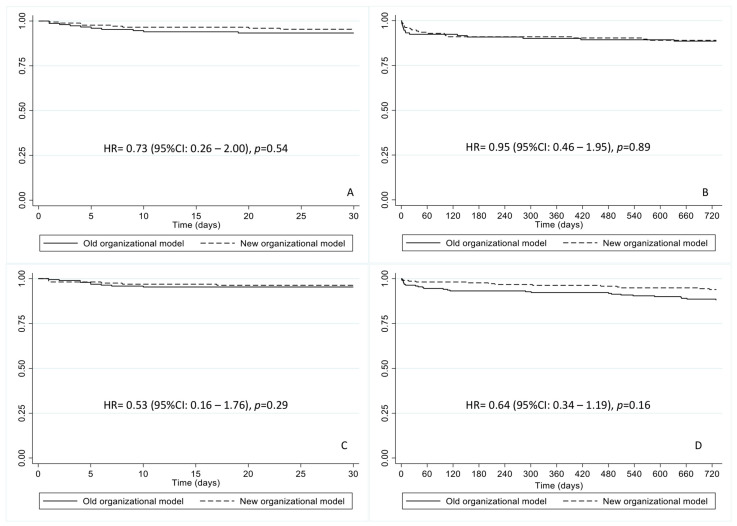
(**A**) 30-day survival for patients with a diagnosis of STEMI given in the Trento city territory. (**B**) 2-year survival for patients with a diagnosis of STEMI given in the Trento city territory. (**C**) 30-day survival for patients with a diagnosis of STEMI given elsewhere in the region. (**D**) 2-year survival for patients with a diagnosis of STEMI given elsewhere in the region. (HR = hazard ratio).

With regard to 30-day and 2-year mortality, despite a numerical reduction in hazard ratios in deaths for both Trento and the rest of the region with the new organizational model with respect to the old one, no statistically significant differences were achieved (Figure 1, panels a–d). Crude numbers and percentages of mortality outcomes are reported in Table 2, and showed no statistical differences.

## 5. Discussion

As stated in the most recent European Society of Cardiology (ESC) Guidelines on the management of acute myocardial infarction in patients presenting with ST-segment elevation, treatment delays to primary PCI are the most easily audited index of quality of care in STEMI in a “hub and spoke” network, and a reduction in system delay should be addressed to reduce total ischemic time [5]. System delay, in particular, has proved to be independently related to long-term mortality (HR 1.22 for each hour in a study by Terkelsen et al. [10]).

Based on the orographic characteristics of the Trentino region, helicopter flight is of paramount importance to keep system delays as low as possible. Up to 30 June 2013, helicopter flight for patient transportation was allowed only from sunrise to sunset, while only ambulances were allowed by night. Since 1 July 2013, after adaptation of structures and training of the personnel, helicopter rescue service was the preferred mode of transportation for patients requiring primary PCI for STEMI. However, it should be noted that helicopter flight is not allowed in the case of dense fog, storms, or snowfalls, thus affecting our results.

Implementation of night flight required a significant economic and organizational effort with the aim of guaranteeing equity of access to optimal care to patients irrespective of their place of residence.

This effort has been outweighed by a significant reduction in the treatment delay for patients coming from outside the chief town. Of note, an indication of an increase in the delay in the group of patients diagnosed in the city territory in the same time frame has been observed. The cause of this delay is not clear; it could be related to an overall increase in the number of the procedures (i.e., to a higher probability of operating room occupation) or to a higher complexity of the patients arriving at the catheterization laboratory.

The reduction in the diagnosis-to-reperfusion time is expected to reduce mortality, based on previous findings. In particular, in a large North American registry, a longer door-to-balloon time was associated with higher in-hospital and 30-day mortality [14], and in another study a higher mortality was demonstrated for patients with a door-to-balloon time >90 min [15]. Similarly, a sub-analysis of two randomized trials (CADILLAC and HORIZONS-AMI) found a lower mortality for patients with a door-to-balloon time lower than 90 min (3.1% vs. 4.7%, *p* < 0.001), in particular for high-risk patients [16].

Nevertheless, not even the large (more than 95,000 patients) CathPCI registry has shown a reduction in in-hospital mortality despite a reduction in door-to-balloon time (83 min from 2006–2007 vs. 67 min from 2008–2009, *p* <0.001) [11]. Similarly, a significant reduction in mortality was not demonstrated in our study, which also has the limitation of the small sample size. Among the confounding factors that could affect these results, the most important is that the total ischemic time also depends on the time between symptoms onset and emergency system activation (the “patient delay”), which is highly variable. Moreover, the association between door-to-balloon time and mortality has been questioned; low-risk patients could be treated more rapidly than high-risk patients requiring clinical stabilization and the execution of diagnostic exams, resulting in a relevant bias [17,18].

However, an improvement in ST-segment elevation resolution was shown for patients coming from outside the city of Trento; in the literature, these data have been linked to a reduction in short- and long-term mortality and could have other potential clinical benefits, for example, in reducing heart failure [19].

This study carries some limitations. First of all, data have been retrospectively evaluated. Moreover, it is a single-center experience and the sample size is relatively small; therefore, local or even individual conditions could have had a significant impact on the general outcome. The sample size was probably not sufficient to analyze the objectives of the study. On the other side, expanding the time frame of enrollment could have introduced other variables that could affect the results (e.g., different antiplatelet drugs or different techniques or materials used during primary PCI). However, some data should be evaluated with caution, e.g., ST-segment elevation reduction >50% and long-term prognosis were slightly worse for patients in the chief town area.

Nevertheless, these data could be helpful for preliminary evaluation of other time-dependent interventions, e.g., in the setting of stroke for mechanical thrombectomy, and as a support for a deeper analysis including health economics or logistic evaluations.

## 6. Conclusions

The implementation of the night flight proved to be effective in reducing the time between the diagnosis and the treatment of patients in the setting of STEMI, improving ST-segment elevation resolution, although no impact was shown on short- and long-term mortality. Nevertheless, our experience could be helpful for preliminary evaluation of time-dependent interventions (e.g., severe trauma, stroke, etc.), and as a support for a deeper analysis including health economics or logistic evaluations, wherever geographic or orographic limitations to ground transportation are found, for example, in mountainous regions or islands. This could be of paramount importance in guaranteeing equity of access to optimal care to patients irrespective of their place of residence.

## Figures and Tables

**Table 1:** Baseline characteristics according to the two different territorial areas in the old and in the new organizational models.

KERRYPNX	Patients Coming from the Chief Town			Patients Coming from Outside the Chief Town		
Baseline Characteristics	OOM (149 pts)	NOM (174 pts)	*p*-Value	OOM (215 pts)	NOM (213 pts)	*p*-Value
Male sex	110 (72.9)	135 (73.4)	0.91	159 (74.0)	159 (74.7)	0.87
Age	66 (13)	66 (12)	0.81	66 (14)	65 (12)	0.29
BMI	25.7 (23.9–28.1)	25.3 (23.0–28.1)	0.43	25.7 (23.9–27.8)	26.1 (23.7–28.4)	0.56
Family history of CAD	51 (36.7)	62 (37.6)	0.87	82 (39.8)	82 (39.4)	0.94
Diabetes	25 (18.0)	32 (18.7)	0.87	32 (15.2)	34 (16.3)	0.77
Dyslipidemia	69 (50.4)	93 (55.7)	0.35	112 (55.2)	102 (49.5)	0.25
Smoking history	50 (36.2)	53 (31.7)	0.41	63 (30.4)	65 (31.2)	0.86
CKD	4 (2.9)	3 (1.8)	0.50	7 (3.3)	6 (2.9)	0.79
Hypertension	85 (61.1)	96 (56.5)	0.41	121 (57.9)	115 (55.0)	0.55
Previous MI	7 (4.9)	7 (4.1)	0.79	10 (4.7)	12 (5.6)	0.67
Previous PCI	14 (9.9)	21 (12.2)	0.51	15 (7.0)	22 (10.3)	0.22
Previous CABG	4 (2.8)	2 (1.2)	0.41	2 (0.9)	2 (0.9)	1.00
Killip class:						
1 2 3 4	130 (91.6) 4 (2.8) 2 (1.4) 6 (4.2)	151 (87.8) 13 (7.6) 3 (1.7) 5 (2.9)	0.28	201 (93.5) 9 (4.2) 2 (0.9) 3 (1.4)	199 (93.4) 9 (4.2) 1 (0.5) 4 (1.9)	0.98
Anterior MI	66 (48.2)	83 (50.9)	0.64	107 (50.5)	94 (45.6)	0.32
Time between symptoms onset and EMS activation, min	116 (62–204)	100 (57–194)	0.45	108 (52–230)	97 (52–180)	0.41
Day/Time of diagnosis:			0.21			0.29
-Working hours	52 (37.2)	74 (44.8)		101 (47.4)	94 (45.9)	
-Holidays	38 (27.1)	32 (19.4)		44 (20.7)	55 (26.8)	
-Night shifts	50 (35.7)	59 (35.8)		68 (31.9)	56 (27.3)	
Mean of hub hospital arrival			0.74			**0.01**
-Ambulance	64 (48.8)	76 (51.4)		75 (37.0)	60 (25.0)	
-Helicopter Rescue	25 (19.1)	23 (15.5)		128 (63.0)	150 (75.0)	
-Self presentation	42 (32.1)	49 (33.1)		-	-	
Single vessel disease	66 (46.5)	88(51.2)	0.41	66 (46.5)	88(51.2)	0.41
Radial access	114 (80.3)	151 (87.8)	0.07	183 (85.1)	194 (91.1)	0.07

Legend: Pts: patients. OOM: old organizational model. NOM: new organizational model. BMI: body mass index. Q1: first quartile. Q3: third quartile. CAD: coronary artery disease. CKD: chronic kidney disease. MI: myocardial infarction. PCI: percutaneous coronary artery intervention. CABG: coronary artery bypass grafting. Values are expressed as number (%), mean (standard deviation), or median (interquartile range), as appropriate.

**Table 2:** Procedural results and performance indicators according to the two different territorial areas in the old and in the new organizational models.

	Patients Coming from the Chief Town			Patients Coming from Outside the Chief Town		
Procedural Results	OOM (149 pts)	NOM (174 pts)	*p*-Value	OOM (215 pts)	NOM (213 pts)	*p*-Value
ST-segment elevation reduction >50%	58 (40.9)	80 (46.5)	0.31	78 (36.3)	115 (54.0)	<0.01
Final TIMI flow 3	116 (81.7)	134 (77.9)	0.41	171 (79.5)	163 (76.5)	0.45
**Performance indicators**						
DTR time, min	68 (58–88)	75 (60–94)	0.07	105 (82–137)	97 (78–119)	**<0.01**
DTR time during nighttime, min	73 (63–89)	75 (63–93)	0.56	119 (97–146)	100 (87–130)	**0.02**
DTR time ≤120 min	126 (90.0)	151 (91.0)	0.77	135 (64.3)	156 (76.9)	**<0.01**
DTR time ≤90 min	109 (77.9)	118 (71.1)	0.18	69 (32.9)	83 (40.9)	0.09
**Outcome**						
30-day mortality	11 (8.7)	7 (4.7)	0.22	9 (4.7)	7 (4.4)	>0.99
2-year mortality	16 (12.6)	16 (10.7)	0.71	26 (13.5)	17 (10.8)	0.51

Legend: Pts: patients. OOM: old organizational model. NOM: new organizational model. TIMI: thrombolisis in myocardial infarction. DTR: diagnosis to reperfusion. Values are expressed as number (%), mean (standard deviation), or median (interquartile range), as appropriate.

## Data Availability

The data that support the findings of this study are available from the corresponding author, Filippo Zilio, upon reasonable request.
